# Is the Voltage-Dependent Anion Channel a Major Player in Neurodegenerative Diseases?

**DOI:** 10.3390/ijms26136138

**Published:** 2025-06-26

**Authors:** Sebastian Neumann, Rolf Heumann

**Affiliations:** 1 Department of Biochemistry II—Molecular Biochemistry, Faculty of Chemistry and Biochemistry, Ruhr-Universität Bochum, 44801 Bochum, Germany; 2 Department of Biochemistry II—Molecular Neurobiochemistry, Faculty of Chemistry and Biochemistry, Ruhr-Universität Bochum, 44801 Bochum, Germany

**Keywords:** voltage-dependent anion channels, Alzheimer’s disease, Parkinson’s disease, amyotrophic lateral sclerosis, Huntington’s disease

## Abstract

The family of voltage-dependent anion channels (VDACs) comprises three isoforms (VDAC-1, VDAC-2, VDAC-3). VDACs have been extensively described as localised in the outer mitochondrial membrane where they are involved in the exchange of ions, metabolites, and ATP/ADP between mitochondria and cytosol. The VDAC interacts with disease-specific proteins and thus regulates the mitochondrial function and controls the cellular energy resources, explaining its involvement in cell death and apoptosis. In addition, VDAC-1 and -2 can also be found at other cellular locations such as in the sarcoplasmic reticulum, in the endoplasmic reticulum, as well as in the plasma membrane. Through single-channel pore regulation, oligomerisation, or changed expression levels the VDAC is involved in different neurodegenerative diseases such as Alzheimer’s disease, Parkinson’s disease, Amyotrophic lateral sclerosis, Huntington’s disease, and others. Here, we critically summarise current discussions about the VDAC as a common key player for these diseases. We suggest that the VDAC acts as a transmembrane multifunctional regulatory protein which might serve as a pharmacological target for the development of novel drugs against neurodegenerative diseases such as the application of recombinant antibody technology.

## 1. Voltage-Dependent Anion Channel Function and Structure

The purpose of this review is to provide a current overview of the role of all three VDAC isoforms regarding their involvement in neurodegenerative diseases. Although excellent reviews have been published by others previously [1,2,3,4], these reviews often focus either on VDAC-1 or on one selected neurodegenerative disease.

**Figure 1:**
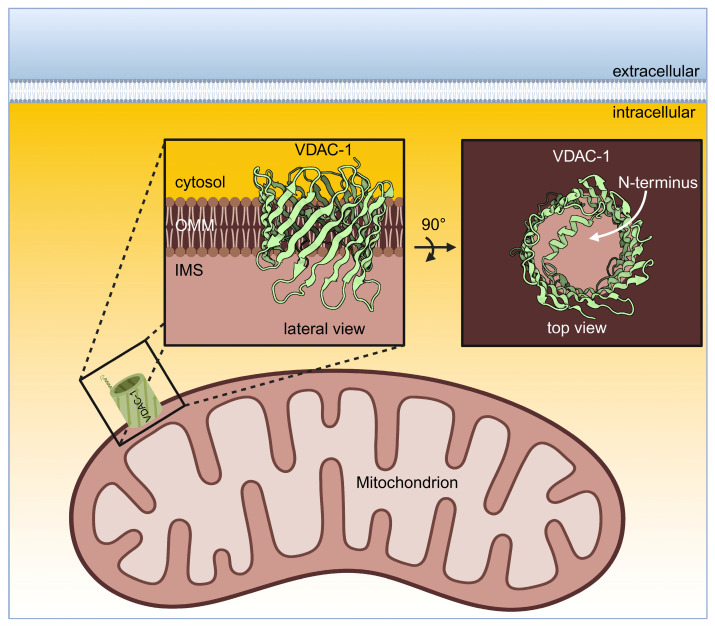
Structure of human VDAC-1. The three-dimensional structure of human VDAC-1 is shown in a mitochondrion. The VDAC-1 is embedded into the outer mitochondrial membrane (OMM) facing the cytosol and the intermembrane space (IMS). Please note, the structure of VDAC-1 is illustrated in a lateral view showing the β-barrel structure (insert image, left) and in a top view enabling the view on the α-helix in the channel lumen (insert image, right). This figure was created in https://BioRender.com/t55l5te (accessed on 19 June 2025) and the entry 2JK4 [20] of the Protein Data Bank was used as a template.

In the year 1976, the voltage-dependent anion channel (VDAC) was discovered in the mitochondria of the unicellular organism *Paramecium aurelia* [5]. Later in 1979, the VDAC was found in mitochondria derived from rat liver [6]. In between, it was found that the VDAC—also named as porin—is expressed in all eukaryotes [7] and comprises a family of the three isoforms VDAC-1, VDAC-2, and VDAC-3 (Table 1) [8]. While the gene coding for VDAC-1 and VDAC-3 has nine exons, the gene for VDAC-2 has ten exons in mammals [9,10]. Furthermore, there are two splice variants for VDAC-1, while there is only one splice variant for VDAC-2 and VDAC-3 [7]. The cDNA sequences of both VDAC-1 and VDAC-2 have a homology of 90%, whereas the one for VDAC-3 is 68% between human and mice. All VDAC genes are encoded in the nucleus; the protein is synthesised at ribosomes in the cytosol and finally imported into the outer mitochondrial membrane (OMM) [11,12,13,14]. The three VDAC isoforms differ in their expression level: VDAC-1 is the most widely expressed form in mammals, followed by VDAC-2, and the lowest expression level was found for VDAC-3 [7]. In addition, the three isoforms vary in their tissue distribution: VDAC-1 and VDAC-2 are expressed in different tissues like brain, heart, liver, and skeletal muscles [15,16], and VDAC-3 shows expression in the liver, lungs, spleen, ovary adrenal gland, and in testes (Table 1) [17]. The molecular weight of the VDAC corresponds to ~30 kDa [18]. In the year 2008, the structures of human and murine VDAC-1 were solved, showing that VDAC-1 is a β-barrel build of 19 anti-parallel β-sheets sloped in a 46° angle and an α-helix at the N-terminal (Figure 1) [9,19,20,21]. Regarding VDAC-2, its structure was determined from zebrafish [22], and there is no three-dimensional structure for VDAC-3 so far.

The pore-forming channel has a diameter of 2.5 nm in its open state [9]. As the VDAC is located in the OMM, it exchanges ions and metabolites, as well as adenosine triphosphate (ATP) and adenosine diphosphate (ADP), between the cytosol and mitochondria [23]. The inner mitochondrial membrane (IMM) contains many carrier proteins and transporters for the specific transport across the IMM [24]. The VDAC shows a certain ion selectivity such that sodium and potassium ions can pass through the channel [25]. Furthermore, dimers of human VDAC-1 and VDAC-2 function as scramblase-type lipid transporter in the OMM for the transport of phospholipids into the intermembrane space of mitochondria [26]. The transmembrane potential regulates the VDAC activity, which is the reason why these channels are named voltage dependent. When VDAC-1 was inserted into the bilayer lipid membrane in in vitro experiments, the channel had a high conductance associated with an open state enabling free transport of monovalent ions and anionic metabolites at a low membrane potential (±20 mV). When the transmembrane potential was increased or decreased in the range of ±20 to ±40 mV, the VDAC permeability changed so that the Ca^2+^ transport increased while the permeability for monovalent ions, metabolites, and nucleotides was inhibited [15,27,28]. Furthermore, while in vitro experiments using artificial membranes showed high conductance for VDAC-1 and VDAC-2, the human VDAC-3 showed low conductance, and the membrane potential did not influence its activity (Table 1) [29,30]. The N-terminus of the VDAC functions as a voltage sensor and its movement leads to conformational changes of the barrel influencing the pore diameter. The channel selectivity is changed by a decreased pore diameter [30,31]. It is the interaction of the VDAC with cytoplasmic proteins such as hexokinase (HK), tubulin, or α-synuclein (αSyn) preferentially that affects the change from the open to the closed pore rather than changes of the membrane potential in a living cell [3,24,27].

### 1.1. Extramitochondrial Locations of VDACs

Besides the expression of the VDAC in the OMM, the VDAC is detected in various other subcellular locations such as the sarcoplasmic reticulum, endoplasmic reticulum [32], and the plasma membrane (Table 1) [33,34,35]. In mice, the first exon of the VDAC-1 pre-mRNA is alternatively spliced, resulting in two different mRNAs splice variants, one coding for the VDAC-1 located in the mitochondria (mt-VDAC-1) and one coding for the VDAC-1 found in the plasma membrane (pl-VDAC-1). As a result of alternative splicing, the pl-VDAC-1 mRNA codes for a short hydrophobic signal peptide of 13 amino acids, which is required to guide the pl-VDAC-1 via the secretory pathway to the plasma membrane. This signal peptide is cleaved off before the pl-VDAC-1 is inserted into the plasma membrane. Finally, mature mt-VDAC-1 and pl-VDAC-1 have identical amino acid sequences [36]. Although human cells also contain the pl-VDAC-1 in their plasma membrane, this is not the result of alternative splicing, but an alternative mechanism of dynamic membrane protein redistribution was proposed [35]. Alternatively, pl-VDAC-1 was found to function as the receptor for human plasminogen kringle 5 (K5) in the cell membrane of human umbilical vein endothelial cells (HUVECs), and K5 may induce the intracellular translocation of VDAC-1 to the plasma membrane [37]. The amino acid residues of mt-VDAC-1 exposed to the cytosol are found on the extracellular site in the case of pl-VDAC-1 [38,39].

The physiological function of pl-VDAC-1 has not been completely uncovered so far; however, pl-VDAC-1 behaves as an NADH-ferricyanide reductase involved in the normal cellular redox homeostasis [40,41]. Furthermore, pl-VDAC-1 participates in cell volume regulation and in ATP release, as shown in murine cells [42]. Importantly, pl-VDAC-1 participates in apoptosis as described in detail below in 1.3. Interestingly, disease condition can lead to a mistargeting of VDAC-1 to unconventional cellular locations. High concentration of glucose (glucotoxicity) induces VDAC-1 overexpression in insulin-secreting β-cells in type 2 diabetes (T2D) patients. Consequently, VDAC-1 becomes mistargeted into the plasma membrane and there is a loss of ATP for β-cells. The antidiabetic drug metformin prevents glucotoxicity-induced VDAC-1 overexpression, showing that VDAC-1 serves as a target for disease treatment [43]. Other VDAC isoforms, i.e., VDAC-2 and VDAC-3, show extramitochondrial expression as these isoforms were found in the outer dense fibres, which are a cytoskeletal part of the sperm flagellum [44]. Furthermore, the plasma membrane or the acrosomal membrane of bovine [45], mouse [46], and human [47] spermatozoa contain VDAC-2 and, at least for human spermatozoa, it was shown that pl-VDAC-2 is involved in sperm function by enabling Ca^2+^ transmembrane transport [47]. Other examples of extramitochondrial regulation are the upregulations of VDAC-1 and VDAC-2 in the plasma membrane of human pancreatic cancer cells [48] and of pl-VDAC-2 upon iron deprivation in erythroleukemia K562 cells [49]. The increased expression of pl-VDAC-2 might be explained by a compensatory mechanism because iron deprivation induces hypoxia, which in turn leads to peroxidation of membrane lipids [50]. VDAC-mediated transport becomes activated by membrane lipid peroxidation. In addition, the activity of the enzymes aldolase A as well as copper and zinc containing superoxide dismutase (CuZnSOD) controlling the redox status can be activated by VDAC-2 [49,51].

### 1.2. VDACs and Mitochondrial Dynamics

Mitochondria are considered dynamic organelles because of their proliferation by fusion and fission, transport processes, and selective degradation [52,53]. VDAC-mediated mitochondrial dynamics result from the interaction with proteins required for fusion like mitfusins and those needed for fission such as dynamin-related proteins (Drp-1) [54]. Furthermore, mitochondrial fusion and fission become affected by altered VDAC expression or activity. Whereas high activity of the VDAC leads to mitochondrial depolarisation and fragmentation, low VDAC activity may promote fusion and maintenance of a healthy mitochondrial network [55]. The selective degradation of damaged or dysfunctional mitochondria—called mitophagy—is regulated by involving the VDAC due to its interactions with proteins such as phosphatase and tensin homologue (PTEN)-induced putative kinase protein 1 (PINK1) and parkin. Furthermore, mitophagy can be impaired by a dysregulated VDAC activity resulting in accumulation of dysfunctional mitochondria [56]. Additionally, VDAC contributes to cellular signalling pathways targeting kinases, phosphatases, as well as cytoskeletal components influencing mitochondrial dynamics [57,58]. Consequently, VDAC dysregulation affects signalling, leading to disturbed mitochondrial dynamics [59,60]. Overall, mitochondrial dysfunction in several diseases might be better understood by investigating the influence of the VDAC on mitochondrial dynamics.

### 1.3. The Involvement of the VDAC in Apoptosis

The development of neurodegenerative diseases is in many cases associated with mitochondrial dysfunction along with caspase-mediated apoptosis [61,62]. Due to this and to point out the role of VDAC regarding apoptosis, the molecular steps of apoptosis are shortly summarised here. The programmed cell death called apoptosis can be divided into the extrinsic pathway and the intrinsic mitochondrial-mediated pathway. External ligands binding to cell surface death receptors like death receptor 4 and 5 (DR4, DR5), tumour necrosis factor (TNFα), TNF-related apoptosis-inducing ligand (TRAIL) receptors, tumour necrosis factor receptor 1 and 2 (TNFR1, TNFR2), and Fas/CD95 activate these receptors. Upon activation, the death signalling complex (DISC) is formed, which activates pro-caspase-8, that in turn activates caspase-3 and -7. Finally, this executes the cell death [63,64].

Intracellular signals such as elevated levels of reactive oxygen species (ROS), DNA damage, Ca^2+^ overload, and chemotherapy drugs result in the activation of the intrinsic mitochondrial-dependent apoptosis. These apoptotic signals change the permeability of the OMM, releasing the apoptotic factors cytochrome *c* (Cyto *c*) and apoptosis-inducing factor (AIF) into the cytosol. A two-step mechanism is required for the release of Cyto *c* [65]. In the IMM, Cyto *c* forms a complex with the phospholipid cardiolipin (CL) [66]. CL is exclusively found in mitochondria, mainly in the IMM [67]. Besides its pleiotropic functions, it anchors the proteins of the electron transport chain (ETC) and Cyto *c* in the IMM [68]. Under apoptotic conditions such as high level of ROS, CL can translocate from the IMM to the OMM. Thereby, free movable CL can bind to Cyto *c* converting it into a peroxidase, catalysing the oxidation of CL, and the peroxidation reaction enables the dissociation of Cyto *c* form the Cyto *c*/CL complex [69]. This results in the liberation of Cyto *c* from the IMM into the intermembrane space (IMS). In a second step, Cyto *c* escapes into the cytosol through a pore in the OMM. Such a pore can be built of Bax [65], Bak, or complexes including the VDAC [70], such as the second mitochondria-derived activator of caspase (SMAC) or the human ortholog direct IAP-binding protein with a low pI (Diablo), endonuclease G (EndoG), and high-temperature requirement protein A2 [71,72]. Cell death proteases (caspases) are activated by these released factors. Cyto *c* interacts with apoptotic protease-activating factor 1 (Apaf-1), forming an oligomeric structure in the presence of dATP. The apoptosome is built upon the binding of this oligomeric structure with cytoplasmic inactive pro-caspase-9. Thereby, pro-caspase-9 becomes activated to caspase-9 that in turn cleaves pro-caspase-3 and -7, obtaining activated effector caspase-3 and -7. The cell is destroyed from within by these activated effector caspases by cleaving cellular substrates and thereby forming apoptotic bodies [71]. Calpains or cathepsins cleave AIF, which translocases to the nucleus for activating chromatin degradation and condensation. Furthermore, chromatin DNA is cleaved into nucleosomal fragments by EndoG, which is transferred into the nucleus as well. From mitochondria into the cytosol, released SMAC antagonises caspase inhibition by interacting with inhibitor of apoptosis proteins (IAPs) [73]. In addition, caspase-8 mediates crosstalk between the extrinsic apoptotic pathway and the intrinsic mitochondrial-mediated apoptotic pathway by cleaving Bid, resulting in the truncated protein tBid which is transferred to the mitochondria for activating the intrinsic apoptosis [74]. The apoptogenic proteins cross the OMM via several possible mechanisms. One possibility is the rupture of the OMM, enabling non-specific release of apoptotic initiators out of the mitochondrial intermembrane space [75]. Furthermore, the permeability transition pore (PTP) opens upon Ca^2+^ overload or after overproduction of ROS [76]. Proteins of the B cell lymphoma 2 (Bcl-2) family can be differentiated into anti-apoptotic proteins such as Bcl-2 and Bcl-xL and pro-apoptotic proteins like Bak, Bax, Bid, and Bim [77]. An additional mechanism describes how large channels are built up by Bax and/or Bak oligomers [78,79], hetero-oligomers of Bax and VDAC-1 [80,81], or oligomers of VDAC-1 [82,83,84,85,86,87,88,89,90,91,92,93]. Additionally, apoptosis can be regulated by the interaction of VDAC-1 with anti-apoptotic proteins providing a further level of control over apoptosis [87,94,95]. The interaction of Bcl-2 and Bcl-xL with VDAC-1 mediates protection from apoptosis [96,97]. In detail, the Bcl-2 homology 4 (BH4) domain of Bcl-2/Bcl-xL is sufficient and essential for interacting with VDAC-1 and thereby inhibiting apoptosis [98]. VDAC-1 overexpression induces apoptosis in all investigated cell types ranging from human to animal, fish, and plant cells [89,99,100,101,102,103]. Consequently, VDAC-1 inhibitors such as 4-acetamido-4-isothiocyanato-stilbene-2,2-disulfonic acid (SITS), 4,4′-diisothiocyanostilbene-2,2-disulfonic acid (DIDS), 4,4′-diisothiocyanatodihy-drostilbene-2,2′-disulfonic acid (H_2_DIDS) or ruthenium red (RuR) prevent apoptosis by their direct interaction with VDAC-1 [90,100]. In line with this, apoptosis can also be inhibited by the overexpression of Bcl-2 as well as hexokinase 1 (HKI) as these are anti-apoptotic proteins [99,104]. Accordingly, using non-small cell lung cancer cells, cisplatin-induced apoptosis was prevented due to siRNA-mediated reduction in levels of VDAC-1 expression [105].

Besides the involvement of the mitochondrial VDAC in apoptosis, the plasmalemmal VDAC is also involved in apoptosis. The pl-VDAC-1 contributes to apoptosis as demonstrated in neuronal cells such as the human neuroblastoma cell line SK-N-MC, in the mouse hippocampal cell line HT22, and in primary differentiated hippocampal neurons which were stimulated with the protein kinase inhibitor staurosporine. These conditions lead to the activation and opening of pl-VDAC-1 preceding the activation of caspases. The extracellular application of anti-VDAC antibodies inhibited apoptosis in these experiments, demonstrating the involvement of pl-VDAC-1 [106,107]. In line with this, extracellularly applied anti-VDAC antibodies mediated protection from amyloid β peptide-induced apoptosis in HT22 cells, and in the mouse cholinergic septal neuronal cell line SN56 [108] and from 15-deoxy-Δ^12,14^-prostaglandin J2 (15d-PGJ_2_)-induced apoptosis in rat primary cortical neurons [109]. Furthermore, a study using the prostate cancer cell line LNCaP led to the suggestion that pl-VDAC-1 might be a positive stimulator of the extrinsic apoptotic pathway [110,111].

### 1.4. Involvement of pl-VDAC-1 in Neuroprotection

For investigating the role of the small GTPase Rat sarcoma (Ras) in a neuronal context, a genetically modified mouse was created, in which human constitutive activated V12-Ha-RAS is expressed in postmitotic neurons, thereby over-activating the endogenous downstream mitogen-activated protein kinase (MAPK) [112]. Several studies confirmed that neurons such as facial motoneurons, the substantia nigra, cortex and hippocampus were protected from chemical or mechanical insults [112,113,114,115,116,117]. However, the detailed molecular mechanism explaining the neuroprotection remained elusive. A proteome study showed that in mice expressing constitutively activated Ras in neurons (=transgenic activation of neuronal Ras), several proteins were changed in their expression level, i.e., the mitochondrial respiratory chain, synaptic protein recycling, and vesicle-mediated transport were upregulated while microtubulin remodelling proteins and VDAC-1 were downregulated.

**Figure 2:**
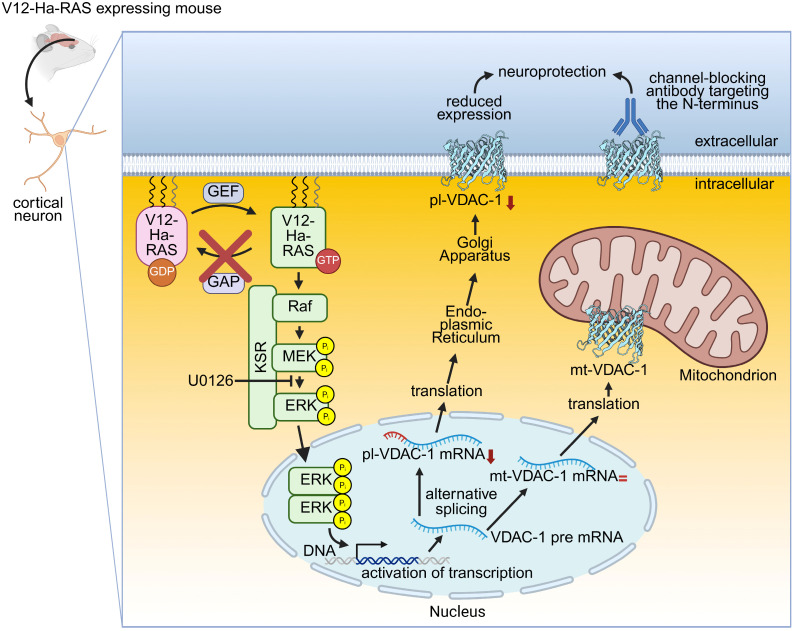
Schematic outline for the involvement of pl-VDAC-1 in neuroprotection in a mouse model expressing human V12-Ha-RAS in postmitotic neurons. Ha-RAS is anchored to the cytoplasmic membrane by two palmitoyl (black) and one farnesyl (grey) anchors. Guanine–nucleotide exchange factors (GEF) activate RAS by the exchange of GDP to GTP. V12-Ha-RAS is locked in its GTP-bound state because it cannot be inactivated by GTPase-activating proteins (GAP) due to the V12 mutation. V12-Ha-RAS then permanently activates the mitogen activated protein kinase (MAPK) signal transduction via rapidly activated fibrosarcoma (RAF), MAPK/ERK kinase (MEK), and extracellular-signal-regulated-kinase (ERK). MEK promotes BRAF activation through allosteric control of KSR proteins, with a kinase suppressor of RAS (KSR) serving as a molecular scaffold for effective signalling. Phosphorylations are indicated by yellow circles with P_i_. MEK can be inhibited by U0126, resulting in a blockage of the downstream signalling. In the nucleus, a dimer of activated ERK stimulates the transcription of certain genes. The VDAC-1 mRNA becomes alternatively spliced into pl-VDAC-1 mRNA and mt-VDAC-1 mRNA. The level of pl-VDAC-1 mRNA is selectively decreased by the activation of the RAS/MAPK pathway in comparison to wild-type mice. In mouse, the pl-VDAC-1 requires a signal peptide guiding the VDAC-1 to the plasmalemmal membrane; this sequence is schematically outlined in red in the pl-VDAC-1 mRNA. Upon translation, the mt-VDAC-1 is inserted into the outer mitochondrial membrane (OMM), while the pl-VDAC-1 is passed by the endoplasmic reticulum and the Golgi apparatus and directly inserted into the cell membrane. In this mouse model, a reduced expression of pl-VDAC-1 causes neuroprotection, which is phenocopied by the extracellular application of an anti-VDAC-1 antibody, resulting in channel blocking. The first 100 N-terminal amino acids of VDAC-1 are the target of the used anti-VDAC-1 antibody. Mt-VDAC-1 and pl-VDAC-1 are shown in their three-dimensional protein structure (3EMN; [21]). Black thin arrows guide through different steps, while small dark red arrows pointing downwards represent decreased mRNA level or decreased protein expression level, respectively. An unchanged mRNA level is indicated by a dark red equal sign. A T-ending arrow indicates inhibition. The meaning of the arrows remains the same for all subsequent figures. This figure was created in https://BioRender.com/wxdvjtc (accessed on 19 June 2025) in a modification from [118].

Focusing on VDAC-1 demonstrated a selective decrease in the expression level of pl-VDAC-1, while mt-VDAC-1 was unchanged in the cortex and hippocampus of this mouse model. Furthermore, the selective decrease in pl-VDAC-1was confirmed in primary cortical cultures derived from this mouse model. The selective inhibition of the MAPK signalling by the inhibitor U0126 led to an attenuated pl-VDAC-1 level in transgenic neurons like in wild-type derived neurons demonstrating that activated V12-Ha-RAS/MAPK signalling influences the alternative splicing of VDAC-1 mRNA. Correspondingly, the extracellular application of a mono-clonal anti-VDAC-1 antibody prior to an excitotoxic glutamate stimulation protected wild-type primary cortical cultures to the same extent as the transgenic V12-Ha-RAS activity in primary cortical cultures (Figure 2). This anti-VDAC-1 antibody targets the first 100 N-terminal amino acids of VDAC-1, including the α-helical part. Taken together, the selective reduction in the expression of pl-VDAC-1 is involved in the V12-Ha-RAS-mediated neuroprotection observed in this mouse model [118].

## 2. Alzheimer’s Disease and VDACs

Typical hallmarks of Alzheimer’s disease (AD) are a decline in cognitive function and progressive memory loss. On the molecular level, AD is characterised by amyloid plaques consisting of amyloid β (Aβ) and neurofibrillary tangles which consist of abnormal hyperphosphorylated tau protein. Tau is required in microtubule stabilisation and furthermore, its association with synaptic loss and cognitive impairments is seen in AP patients. Although the biological mechanisms resulting in sporadic forms of AD are not fully understood so far, it is proposed that cholinergic dysfunction, mitochondrial dysfunction, inflammation, tau accumulation, Aβ plaque formation, inflammatory response, lysosomal dysfunction, and hormone regulation are involved [119].

In early stages of AD pathogenesis, mitochondrial dysfunction is observed with several pathophysiological events such as disruption of Ca^2+^ homeostasis, reduced metabolism, lipid peroxidation, an increase in ROS production, and finally apoptosis. Interestingly, this impaired brain metabolism develops several decades before dementia becomes obvious [120,121,122,123,124]. The accumulation of Aβ and dysfunctional mitochondria can result in oxidative stress and increased production of ROS [125]. High levels of ROS affect mitochondrial components like membrane lipids, mitochondrial DNA (mtDNA), and components of oxidative phosphorylation [126,127]. Furthermore, ROS can oxidise the VDAC, making it dysfunctional [128,129,130].

AD postmortem patient brains showed high levels of VDAC-1 expression in neurites of Aβ deposits, which was also observed in amyloid precursor protein (APP) transgenic mice [131,132,133]. Neurons of AD brains show characteristics of apoptosis and the massive loss of neurons in AD is caused by apoptosis [92,134,135]. As mentioned above (see 1.3), VDAC-1 overexpression triggers apoptosis and this might be one of the reasons for the neuronal cell death in AD.

**Figure 3:**
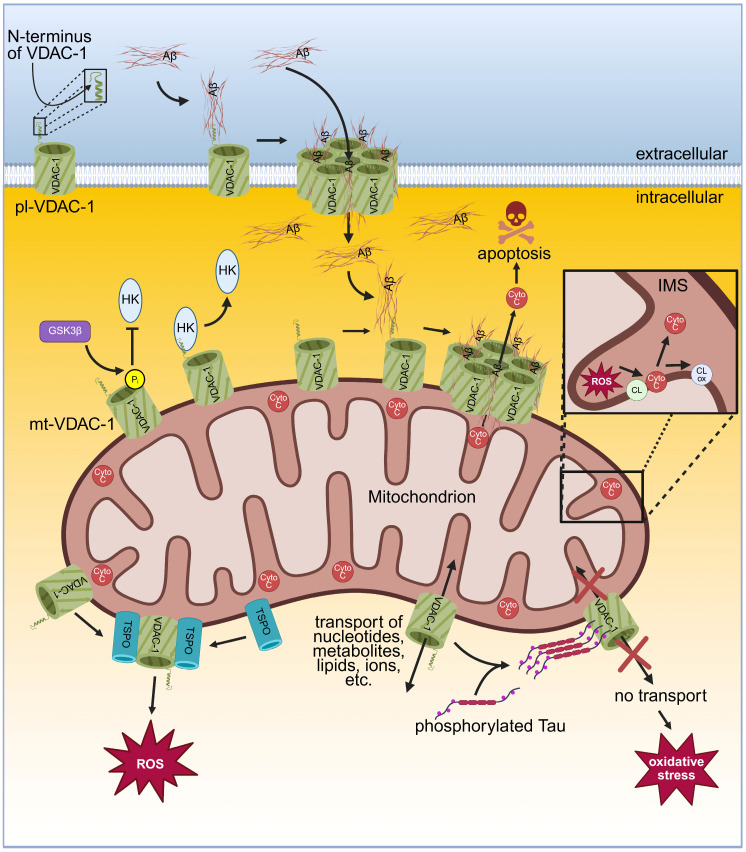
Schematic outline for the role of VDAC-1 in AD. Extracellular amyloid β (Aβ) interacts via the GXXXG motif with the N-terminus of pl-VDAC-1 by electrostatic interactions, as Aβ provides negative charges, and the N-terminus has positive charges. A hetero-oligomer of pl-VDAC-1 and Aβ is formed, allowing the entrance of Aβ into the cytosol. Intracellularly, mt-VDAC-1 interacts with Aβ, forming a hetero-oligomer of mt-VDAC-1, with Aβ enabling the release of cytochrome c (Cyto *c*), resulting in apoptosis. As demonstrated in the insert image on the right side, Cyto *c* gains the property of a peroxidase under apoptotic conditions such as a high level of reactive oxygen species (ROS), resulting in the oxidation of the phospholipid cardiolipin (CL). Oxidised CL (CL ox) has less binding affinity to cytochrome c, leading to its liberation into the intermembrane space (IMS). Aβ can induce the detachment of hexokinase (HK) from mt-VDAC-1. Furthermore, glycogen synthase kinase 3 (GSK3β) can phosphorylate mt-VDAC-1 and thereby inhibits the binding of HK to mt-VDAC-1. In addition, phosphorylated tau can interact with mt-VDAC-1, leading to the closure of the channel with the consequence that there is no further transport of nucleotides, metabolites, lipids, ions, etc. Additionally, mt-VDAC-1 and translocator protein (TSPO) form a complex contributing to ROS. This figure was created in https://BioRender.com/o52n8qk (accessed on 19 June 2025).

Experiments in the HT22 cell line and in the mouse cholinergic septal neuronal cell line SN56 showed that the application of anti-VDAC antibodies protected from Aβ-peptide-mediated neurotoxicity, demonstrating the involvement of plasmalemmal VDAC in AD [108]. Furthermore, pl-VDAC-1 interacts with membrane-related isoform of oestrogen receptor α (mERα), forming a complex in the caveolae [108]. The pl-VDAC along with mERα builds a complex with scaffolding protein caveolin-1 in the caveolae of the human hippocampus and cortex. AD brains show an accumulation of the VDAC in the caveolae of dystrophic neurites of senile plaques [133]. Both the mitochondrial and the plasmalemmal VDAC-1 are involved in Aβ-mediated neurotoxicity according to the following model: extracellular Aβ oligomers interact with the N-terminus of the pl-VDAC-1. The interaction of VDAC-1 with Aβ might involve positive charges of VDAC-1 in its N-terminal domain, negative charges in Aβ, hydrophobic interaction, electrostatic interaction, and interactions via the amino acid sequence GXXXG, where X represents any amino acid in this motif. VDAC-1 provides one GXXXG motif in its α-helix at the N-terminus [136] and Aβ contains three GXXXG motifs [137]. There is the hypothesis that the GXXXG motif of Aβ interacts with the GXXXG motif of VDAC-1 in AD [138,139]. The interaction of Aβ with pl-VDAC-1 results in VDAC-1 oligomerisation and building a large pore consisting of Aβ/VDAC-1 heteromers [16,92]. Aβ enters the cell through this large pore [16,92]. Despite this one possibility via pl-VDAC-1, the cellular entry of Aβ can occur by endocytosis, through clathrin-mediated endocytosis or by dynamin-dependent endocytosis, as both possibilities are most frequently reported [140]. Endocytosed Aβ permeabilises the lysosomal membrane, resulting in its release into the cytosol [141]. Intracellular Aβ interacts with the mt-VDAC-1, resulting in detachment of HKI and induction of VDAC-1 oligomerisation, along with Aβ forming large heteromeric Aβ/VDAC-1 pores. These pores enable Cyto *c* release into the cytoplasm and thereby induce apoptosis (Figure 3) [92]. Consequently, a reduced VDAC-1 expression, as shown in a mouse model, protects from degenerative changes [142]. Additionally, siRNA-mediated silencing of VDAC-1 expression inhibited Aβ entry into the cytosol and protected from Aβ-induced toxicity [92]. Furthermore, as the newly developed small molecule VBIT-4 inhibits VDAC-1 [90], VBIT-4 prevented Aβ-induced VDAC-1 overexpression and apoptotic cell death in neuronal cultures. In addition, using an AD mouse model with VDAC-1 overexpression in neurons surrounded by Aβ plaques, application of VBIT-4 protected from pathophysiological changes such as neurometabolic dysfunction, neuroinflammation, and neuronal cell death. Interestingly, behavioural assessments of this mouse model showed that VBIT-4 prevented cognitive decline. With respect to AD treatment, VDAC-1 is an interesting target and VBIT-4 might be an encouraging drug candidate [143]. However, a recent study showed that VBIT-4 might be toxic to healthy cells, at least as demonstrated for breast adenocarcinoma (MCF-7) cells. High concentration of VBIT-4 induced the suppression of mitochondrial respiration, increased the H_2_O_2_ and ROS production, and led to cell death, thus demanding cautionary application in clinical settings [144].

There is another possible link between AD and pl-VDAC-1: AD patients show an increased formation of prostaglandin D_2_ (PGD_2_) [145], and its metabolite 15-deoxy-Δ^12,14^-prostaglandin J2 (15d-PGJ_2_) exerts a high neurotoxicity [146]. The pl-VDAC-1 was found as a membrane target for 15d-PGJ_2_ [109]. Extracellular application of anti-VDAC-1 antibodies protects from 15d-PGJ_2_-mediated neurotoxicity in rat primary cortical neurons [109].

Recently, it was shown that dimers of VDAC-1 with VDAC-2 function as a phospholipid scramblase [26]. Apoptotic cells lose their lipid asymmetry because phosphatidylserine (PS) becomes externalised to the external leaflet [147]. This externalisation of PS could be explained by the phospholipid scramblase activity of dimeric VDAC in the plasma membrane under apoptotic conditions, as proposed by Rockenfeller [148]. In the context of AD, the author extended this hypothesis, proposing that VDAC dimers could insert APP into the plasma membrane [148].

An additional link emerges from increased glycogen synthase kinase 3 (GSK3β) in AD, resulting in a non-characteristic APP processing, leading to an elevated Aβ production and hyperphosphorylated tau [149]. Furthermore, GSK3β phosphorylates mt-VDAC-1, causing the detachment of HK, which alters the cellular metabolism (Figure 3) [150] by reducing the ATP supply for glycolysis and glucose metabolism, making the cells more prone for apoptosis. As mentioned above, HK detachment can also be induced by Aβ, resulting in VDAC-1 oligomerisation, which enables the release of Cyto *c* and subsequent activation of apoptosis [92].

In AD, an irregular hyperphosphorylation of the tau protein was found, which results in its aggregation and the formation of neurofibrillary tangles [151]. Phosphorylated tau interacts with VDAC-1, as found in the brains of AD mice and AD patients [131,152]. The mitochondrial function is influenced by the interaction of phosphorylated tau with the VDAC [153]. In AD, phosphorylated tau protein binds to the VDAC and thereby closes the channel, yielding impaired transport of ions and metabolites across the mitochondrial membrane (Figure 3) [59,152]. This decreases the ATP production and causes oxidative stress, contributing to neurodegeneration and cognitive decline [154,155]. Interestingly, a VDAC-1 heterozygous mouse model (VDAC-1^+/−^) showed reduced mRNA levels for AD-related genes such as Aβ, APP, and tau, demonstrating that a reduced VDAC-1 expression level protects from AD-mediated neurotoxicity [142].

Furthermore, in AD, VDAC-1 interacts with the translocator protein (TSPO) which is located in the OMM. TSPO is an 18 kDa protein participating in several process such as mitochondrial metabolism, cholesterol import, cell proliferation, inflammation, oxidative stress, and apoptosis [156]. It forms a complex with mt-VDAC-1 (Figure 3), which could probably occur via GXXXG motifs as the N-terminus of VDAC, containing one GXXXG motif (see above) and TSPO, providing three GXXXG motifs [157]. The complex of VDAC-1 with TSPO promotes the overproduction of ROS [157]. Interestingly, TSPO is overexpressed as well (as described for VDAC-1) in the brains of AD patients [158]. In addition, overexpression of TSPO is seen in all AD models [159,160,161,162,163,164].

Post-transcriptional gene expression regarding various cellular processes, including neuronal function, can be regulated by small noncoding RNAs called microRNAs (miRs) [165,166]. The VDAC-1 expression level can be regulated by several miRNAs [167,168,169,170], while specifically miR-29a showed an association with AD [171]. The analysis of postmortem brains from patients suffering from sporadic AD showed a loss of miR-29a [172]. In mice, the knockdown of miR-29 in the brain resulted in massive cell death in the hippocampus and cerebellum, which was explained by an increased VDAC-1 expression level. In miR-29 knockdown cells, apoptosis could be partly inhibited by the downregulation of VDAC-1 expression. The authors draw the conclusion that VDAC-1 expression levels can be explained by miR-29, which plays an important role regarding the cellular survival of neurons in the brain [171]. However, a miR-29 knockdown may influence more targets than just VDAC-1, and miR-29 controls cell survival via VDAC-1 in astrocytes [173]. In conclusion, due to the influence of miRNAs on the expression level of VDAC-1, miRNAs seem to be a promising target for developing therapeutic approaches regarding different neurodegenerative diseases like AD.

Finally, there is a link between AD and T2D involving VDAC. Several correlations between AD and T2D have been described, demonstrating that T2D is a risk factor for AD and vice versa [174,175,176]. Persons with T2D have approximately a 1.5-fold higher relative risk for AD according to a meta-analysis of longitudinal studies [177]. Furthermore, another study showed that the risk of dementia is doubled in T2D patients [178]. The antidiabetic drug metformin was proposed to have a neuroprotective potential because it reduces the risk of AD onset [178]. The neuroprotective potential of metformin can be mechanistically explained by its inhibition of hyperinsulinemia, which in absence of inhibition participates in Aβ plaque formation and yields to the onset of AD [179]. Furthermore, using a tau transgenic mouse model, tau phosphorylation was reduced by metformin, as demonstrated in primary neurons [180]. The connection between AD and T2D is further strengthened by the analysis of postmortem brains, showing defective insulin signalling [181,182]. This link led to the suggestion that AD could be considered as “type 3 diabetes” [183,184]. Diabetes induces changes in insulin signalling, glucose metabolism, vascular function, and modifying Aβ/tau metabolism, resulting in neurodegeneration [185,186,187]. A common feature in AD and T2D is the overexpression of VDAC-1, as found in affected regions of brains from AD patients [131,132,188] and in β-cells of T2D [189,190]. This VDAC-1 overexpression mediates apoptosis in AD and T2D. Interestingly, a study used mice to investigate metformin-mediated side effects, showing increased VDAC-1 levels connected to the formation of dimers and trimers of VDAC-1, along with mitochondrial dysfunction in the cortex, and toxic amyloid pre-fibrillar aggregates were directly induced by metformin. In total, these effects increase the risk for the onset of AD [191].

Taken together, plasmalemmal and mitochondrial located VDACs are strongly involved in the pathology of AD because of increased expression levels, their potential to generate oligomers, and due to their interactions with Aβ, GSK3β, phosphorylated tau, and TSPO.

## 3. Parkinson’s Disease and VDACs

Coming after AD, the second most neurodegenerative disease is Parkinson’s disease (PD) [192]. PD is characterised by motor symptoms such as a resting tremor, muscular rigidity, dystonia, postural instability, and dyskinesias and non-motor symptoms such as anxiety, depression, hallucination, hyposmia, fatigue, sleep disorders, cognitive impairment, and diarrhoea [193,194,195,196]. PD arises from several factors like genetics, aging, and environmental factors. The main pathogenic genes of PD comprise α-synuclein, leucine-rich repeat kinase 2 (LRRK2), phosphatase and tensin homologue (PTEN)-induced putative kinase 1, parkin RBR E3 ubiquitin protein ligase (PRKN), DJ-1 (Parkinsonism associated deglycase, PARK7), glucosylceramidase (GBA), and vacuolar protein sorting-35 (VPS35) [197]. Motor symptoms become obvious after a majority (about 70%) of dopaminergic (DA) neurons have died in the substantia nigra pars compacta (SNpc). Consequently, dopamine is depleted in the striatum and results in the impairment of the thalamo-corticobasal ganglia circuits [198,199,200]. The gold standard for the treatment of PD is the administration of the physiological precursor L-3,4-dihydroxyphenylalanine (L-DOPA) to substitute the striatal dopamine loss. Other pharmacological treatments use inhibitors of catechol-o-methyltransferase (COMT) and monoamine oxidase (MAO)-B, or amantadine. At later stages, another therapeutic option is the deep brain stimulation of the internal part of the globus pallidus (GPi) or of the subthalamic nucleus (STN). Unfortunately, none of the therapeutic options can cure or stop the progression of PD [201]. Furthermore, motor complications develop as a side effect of long-term treatment with L-DOPA, which are referred as L-DOPA-induced dyskinesia [202]. Additionally, the non-motor symptoms cannot be counteracted by L-DOPA.

Pathological hallmarks of PD are the loss of DA neurons and the occurrence of lewy bodies (LBs), which are cytoplasmatic neuronal inclusions consisting mainly of the protein αSyn [203]. Although, the accumulation of misfolded αSyn and its neurotoxic effects and interference with mitochondrial functions have been described in detail in excellent reviews such as [204], we will shortly summarise the key aspects here.

αSyn misfolds by a not fully understood process into amyloid fibrils, which accumulate intracellularly [141]. The small 14 kDa protein αSyn is ubiquitously expressed, especially at presynaptic terminals. The N-terminal of αSyn forms an α-helix structure, enabling the interaction with lipid membranes [205]. The central domain of αSyn is built of a highly hydrophobic motif, which is required for the aggregation of αSyn [206]. The C-terminus of αSyn consists of proline residues and negatively charged amino acids in a region enabling various post-translational modifications such as phosphorylation at serine 129 (S129), which was first identified in extracts from LBs of PD patients [207]. The phosphorylation of αSyn at S129 influences the interaction between αSyn and lipid membrane and strengthens its binding to metal ions and further proteins, resulting in protein aggregation [208]. Pathological misfolding of αSyn starts with the acquirement of a structure rich in β-sheets, followed by self-assembly into intermediates of metastable oligomers, which finally accumulate as fibrils within LBs [209]. Depending on the disease stage, αSyn aggregates were found in different brain regions of PD patients, leading to the proposal that αSyn might transmit from cell to cell via a mechanism like prions [210,211]. The neurotoxicity of αSyn oligomers is mediated by modifying biological membranes in their stability and by interaction with mitochondrial proteins, which become influenced in their function [212]. Consequently, toxic effects are triggered by αSyn oligomers in a cascade such as membrane permeabilization, mitochondrial dysfunction, increased Ca^2+^ influx, oxidative stress, loss of proteostasis, and finally cell death [213,214,215]. Mitochondrial dysfunction is accompanied by the transition of monomeric αSyn to pathological oligomers [204].

**Figure 4:**
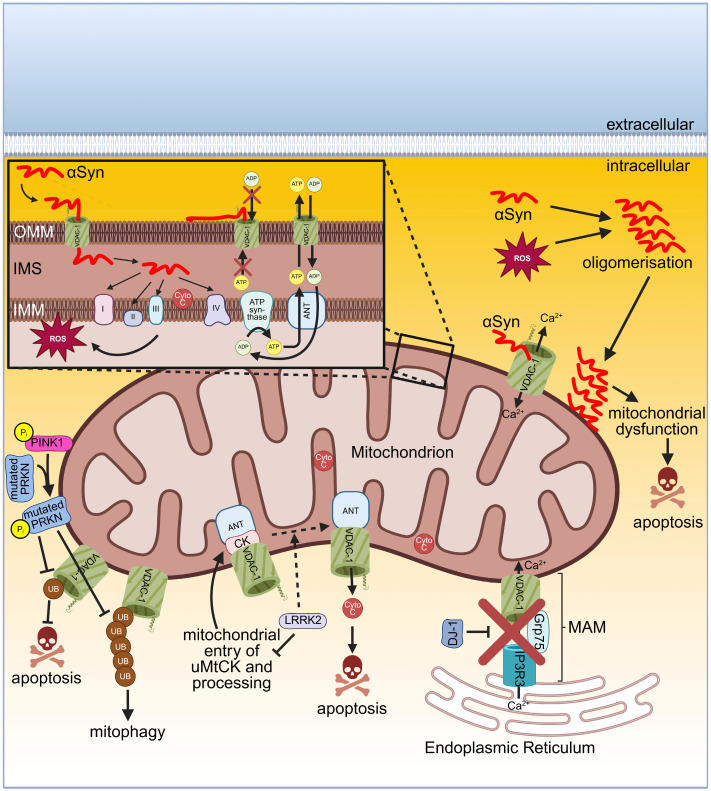
Schematic layout for the role of VDAC-1 in PD. Monomeric α-synuclein (αSyn) can translocate through the mt-VDAC-1 into intermembrane space (IMS), where it directly interferes with complexes I, II, III, and IV of the electron transport chain (ETC) in the inner mitochondrial membrane (IMM), resulting in increased reactive oxygen species (ROS) production. Furthermore, monomeric αSyn can bind into the pore of mt-VDAC-1, blocking the transfer of ADP and ATP and thereby depleting the ATP synthase. The Ca^2+^ ion permeability of VDAC-1 changes upon binding of monomeric αSyn. Under the influence of ROS, monomeric αSyn oligomerises, and these oligomers of αSyn can attach to the outer mitochondrial membrane (OMM), which induces mitochondrial dysfunction leading to neuronal cell death. At the mitochondria-associated membrane (MAM), the inositol 1,4,5-trisphosphate receptor (IP3R3)—glucose-regulated protein 75 (Grp75)—VDAC-1 complex requires DJ-1, and this complex enables the interorganelle transfer of Ca^2+^ ions between the endoplasmic reticulum and mitochondria. In PD, mutant DJ-1 L166P acquires a reduced interaction with the IP3R3-Grp75-VDAC-1 complex and in sporadic PD patients shows a lower level of DJ-1, resulting in disturbed interorganelle Ca^2+^ transport. Leucine-rich repeat kinase 2 (LRRK2) can inhibit the mitochondrial entry of ubiquitous mitochondrial creatine kinase (uMtCK). Normally, uMtCK enters the mitochondria, becomes processed to creatine kinase (CK), and interacts with adenine-nucleotide translocator (ANT) and mt-VDAC-1. Without CK, ANT and VDAC-1 interact with each other, leading to the opening of the permeability transition pore (PTP), yielding to release of Cyto *c*, inducing neuronal apoptosis. At the OMM, phosphorylated phosphatase and tensin homologue (PTEN)-induced putative kinase protein 1 (PINK1) recruits parkin RBR E3 ubiquitin protein ligase (PRKN), which becomes phosphorylated and subsequently ubiquitinates several proteins located in the OMM, among them mt-VDAC-1. While monoubiquitinated VDAC-1 inhibits apoptosis, polyubiquitinated VDAC-1 induces mitophagy. In PD, mutated PRKN impairs ubiquitination of mt-VDAC-1. This figure was created in https://BioRender.com/acoi86y (accessed on 19 June 2025).

Monomeric αSyn directly interacts via its C-terminus with the VDAC. There are two possibilities for the interaction of αSyn with the VDAC which first regulate the permeability of OMM or secondly mediate cytotoxicity. In the first option, monomeric αSyn can bind into the pore of VDAC in the OMM, leading to a sterically blocked channel and thereby inhibiting the flux of ATP and ADP. Consequently, the adenine-nucleotide translocator (ANT) of the IMM is disturbed by a substrate unbalance. This depletes the substrate of ATP synthase and the mitochondrial potential decreases, resulting in an impairment of oxidative phosphorylation. The VDAC blockage by αSyn is reversible, concentration- and voltage-dependent, and might be part of a regulatory mechanism for mitochondrial respiration (Figure 4). Recently, the conformational plasticity of mt-VDAC-2 was demonstrated using αSyn as a cytosolic protein for the investigation of the interaction with mt-VDAC-2, showing a correlation between higher-conductance substates and increased on-rates of αSyn. This led to the suggestion that αSyn perceives dynamic structural variations of VDAC-2 before binding [216]. In the second option, under stress conditions, monomeric αSyn is translocated through the pore of the VDAC into the IMS. There, it can directly interact with the complexes of the ETC embedded in the IMM [217]. αSyn directly targets complex I [215,218,219], complexes II and III [220], and complex IV [221]. The interaction of αSyn with the ETC results in mitochondrial dysfunction due to increased production of ROS (Figure 4). Consequently, monomeric αSyn is oxidised in the cytosol, leading to oligomerisation of αSyn. Oligomeric αSyn associates with the OMM, causing mitochondrial dysfunction and thereby finally mediating neurotoxicity (Figure 4) [217,222]. In addition, the composition of the OMM lipids may influence how αSyn binds to the OMM and thereby influences the complex formation of αSyn with the VDAC, as recently discussed in a review [223]. Furthermore, when αSyn binds to the VDAC, the Ca^2+^ permeability of the VDAC becomes modulated, leading to an increased Ca^2+^ flux through the VDAC (Figure 4) [224]. Comparing the binding affinities between αSyn and VDAC-1 or VDAC-3, respectively, showed a 10-to-100-fold lower affinity to VDAC-3 than to VDAC-1 [225]. The adeno-viral-mediated overexpression of αSyn in the substantia nigra of rat brains resulted in neuronal death of dopaminergic neurons due to an interaction of αSyn with mt-VDAC-1 and its subsequent activation of PTP [226]. In agreement with this, the analysis of a transgenic mouse model which expresses human αSyn A53T shows a PD-like phenotype and revealed that αSyn A53T interacts with neuronal mt-VDAC and with the PTP modulator cyclophilin D [227]. Remarkably, the accumulation of αSyn in the nigral neurons of postmortem PD patients induced a decreased expression level of VDAC-1 compared to the age-matched control group. This could be confirmed in a rat model by the viral-mediated expression of human mutant αSyn A30P leading to a decreased VDAC-1 level in striatal fibres and nigral neurons [228]. The outcome of an altered dopamine homeostasis was investigated using the human neuroblastoma cell line SH-SY5Y and showed reduced VDAC-1 and VDAC-2 protein levels while their mRNA levels remained unchanged, suggesting a dopamine-induced increased protein degradation mediated by mitochondrial proteases. The authors also found a reduced VDAC-3 level but they focused on VDAC-1 and VDAC-2, arguing that in general VDAC-3 shows a low expression level in SH-SY5Y cells [229]. Similar results were observed using the human neuroblastoma cell line NMB as the administration of dopamine induced apoptosis, along with a reduction on the mRNA level for VDAC-1, VDAC-2, and VDAC-3. Interestingly, transient transfection-mediated expression of the human VDAC or human VDAC-2 protected from dopamine-mediated neurotoxicity in NMB cells [230]. It needs to be further investigated why the toxic effect of a 24 h treatment of dopamine (1 µg/mL) is reduced after transfection of cells with the VDAC or VDAC-2 while in other degenerative models (see below), the VDAC acts as a pro-apoptotic protein. Certainly, this calls for a critical pre-evaluation of the therapeutic application in PD when targeting VDACs.

Contrastingly, and in line with the notion that the VDAC could be considered as a protein with pro-apoptotic activity, an increased VDAC mRNA and protein level was observed in SH-SY5Y cells upon rotenone treatment. Rotenone is used in cellular PD models as it inhibits mitochondrial complex I, leading to translocation of Cyto *c* [231]. Furthermore, the expression of VDAC-1 is increased upon 1-methyl-4-phenylpyridinium (MPP^+^) [168,232] and 6-hydroxydopamine (6-OHDA) [233] induction in PD cell models. Recently, the 6-OHDA-induced VDAC-1 upregulation could be reduced by administration of vitamin D in a male rat model of PD [234]. Interestingly, there might be a common control for the expression of VDAC-1 and αSyn, respectively, because both mRNAs are targets of the same miR-7 [168,235].

A changed expression of several miRs has been connected to the pathogenesis of PD [236]. The analysis of postmortem PD brain samples showed a reduction in miR-7 in comparison to samples of a healthy control group. Furthermore, using a mouse model for inducing loss of miR-7 resulted in increased expression of αSyn, along with a reduction in nigral DA neurons and reduced striatal dopamine content [237]. In SH-SY5Y cells, miR-7 overexpression reduced VDAC-1 expression and thereby protected the cells from MPP^+^-induced initiation of apoptosis such as Cyto *c* release and calcium efflux [168]. Interestingly, the synthetic cholesterol-like compound olesoxime (cholest-4-en-3-one, oxime, TRO19622) binds into the OMM and thereby inhibits the translocation of αSyn through the VDAC, thus mediating neuroprotection [238,239]. Recently, a small membrane-binding peptide called HK2p mediated neuroprotection by inhibiting the complex formation between VDAC and αSyn. Furthermore, HK2p induces detachment of αSyn from the OMM, resulting in an open state of VDAC, allowing ATP/ADP exchange and restoring the mitochondrial potential [240]. Recently, the role of the antioxidant and anti-inflammatory phytoalexin resveratrol was investigated in the context of VDAC-1 in the pathogenesis of PD. Using the A53T mouse model, the administration of resveratrol resulted in decreased expression of VDAC-1 and αSyn on the protein level in DA neurons. Consequently, the opening of PTP was prevented by resveratrol in DA neurons. Interestingly, there was no change in the expression levels of VDAC-2 and VDAC-3 in DA neurons. Furthermore, resveratrol improved the cognitive and motor abilities in this model, as shown by animal behavioural tests [241].

Another protein involved in PD is LRRK2. As shown in embryonic neuronal precursors (ETNA) cells and in SH-SY5Y cells, overexpression of wild-type LRRK2 and its mutants R1441C, G2019S, and Y1699C mediated mitochondrial-dependent neuronal apoptosis [242]. LRRK2 directly interacts with three proteins of the PTP, such as ANT, VDAC, and ubiquitous mitochondrial creatine kinase (uMtCK), as demonstrated in PC12 cells, which is a pheochromocytoma cell line derived from the rat adrenal medulla. The processing of immature uMtCK can be inhibited by both LRRK2 and its G2019S mutant due to suppressing the translocation of uMtCK into mitochondria and thereby retaining the preprotein uMtCK on the OMM. Furthermore, the expression of wild-type and G2019S LRRK2 supported the interaction between the VDAC and ANT, which is important for the opening of the PTP, allowing the release of Cyto *c* and resulting in neuronal apoptosis (Figure 4) [243]. However, the exact molecular composition of the PTP is still a matter of ongoing research, although this pore has been studied for over 50 years, as recently reviewed in [76].

Mutations in the genes of PINK1 and PRKN cause recessive forms of PD [244,245,246,247]. PINK1 and PRKN are important regarding mitochondrial quality control. Under cellular stress conditions when mitochondria are depolarised, the phosphorylated serine/threonine kinase PINK1 localises at the OMM, recruiting the E3 ligase PRKN to the OMM as well. Upon phosphorylation by PINK1, PRKN ubiquitinates several proteins of the OMM and thereby induces mitophagy, which is a variant of autophagy for removing damaged mitochondria [248,249]. At least VDAC-1 is essential and required for the PINK1/PRKN-induced mitophagy. Furthermore, VDAC-1 becomes polyubiquitylated by PRKN. Several mutations of PRKN impairment induce mitophagy and thereby damaged mitochondria are not removed by mitophagy, enabling the release of Cyto *c* and resulting in apoptosis (Figure 4) [250]. In addition, it was demonstrated that PRKN directly interacts with all isoforms of the VDAC (VDAC-1, -2, and -3) at the OMM-mediating mitophagy [56]. Recently, cryo-electron microscopy was used solving a structure at a 3.1 Å resolution of dimeric human PINK1 bound to a symmetric array of a central VDAC-2 dimer, while each VDAC-2 protein was surrounded by translocase of the outer membrane (TOM)5 and TOM20 [251]. VDAC-1 can become mono- and polyubiquitinated by PRKN. This leads to different physiological outcomes as VDAC-1 monoubiquitination prevents apoptosis while VDAC-1 polyubiquitination induces mitophagy, demonstrating the central role of VDAC-1 in the antagonistically regulated apoptosis and mitophagy in response to the PINK1–PRKN pathway. Interestingly, the mutant PRKN T415N prevents monoubiquitination of VDAC-1 and consequently apoptosis is not inhibited. Nevertheless, polyubiquitination of VDAC-1 by PRKN T415N is not affected. PRKN T415N belongs to one of the different mutations of PRKN found in PD patients [252]. Interestingly, silencing of PINK1 induced mitophagy associated with a changed mitochondrial morphology, enhanced production of ROS, a loss of the mitochondrial membrane potential, and opening of PTP as shown in mouse dopaminergic MN9D cells [253]. In a PD mouse model, the administration of the drug idebenone upregulated the expression of mt-VDAC-1, thus activating PINK1/PRKN-mediated mitophagy so that damaged mitochondria were removed. Consequently, damage of dopaminergic neurons was reduced, and behavioural disorders were improved [254].

The protein DJ-1 is encoded by the gene PARK7, which belongs to several genes associated with familial forms of early-onset PD. DJ-1 functions as a molecular chaperone, redox sensor, antioxidant scavenger, and transcriptional regulator. DJ-1 can be located in the cytosol, in the nucleus, and upon oxidative stress it is translocated to mitochondria, allowing it to develop its pleiotropic functions [255]. Furthermore, DJ-1 is involved in maintaining the function and integrity of the mitochondrial network, controlling the mitochondrial Ca^2+^ homeostasis via the regulation of the interaction between the endoplasmic reticulum (ER) and mitochondria [256]. Moreover, DJ-1 is an essential and important component of the IP3R3-Grp75-VDAC-1 complex consisting of the ER Ca^2+^ channel inositol 1,4,5-trisphosphate receptor (IP3R3), the mitochondrial chaperone glucose-regulated protein 75 (Grp75), and VDAC-1. This complex is located at the mitochondria-associated membrane (MAM), in particular the VDAC-1 in the OMM, while IP3R3 and GRP75 are inserted into the membrane of the ER. This DJ1-IP3R3-Grp75-VDAC-1 complex enables efficient interorganelle transfer of Ca^2+^ [257,258]. The IP3R3-Grp75-VDAC-1 disrupts upon ablation of DJ-1, resulting in reduced interaction of the ER with mitochondria, disturbed Ca^2+^ efflux from the ER, and accumulation of IP3R3 at the MAM (Figure 4). The PD-associated mutant DJ-1 L166P shows reduced interaction in the DJ1-IP3R3-Grp75-VDAC-1 complex. Furthermore, patients of sporadic PD showed reduced levels of DJ-1 in the substantia nigra associated with lower ER–mitochondria interaction and reduced IP3R3-DJ-1 interaction. In summary, mutated or reduced DJ-1 disturbs the association of ER with mitochondria and consequently takes part in the pathogenesis of PD [257].

Taken together, several PD-related proteins such as αSyn, LRRK2, PINK1/PRKN, and DJ-1 directly interact with the VDAC and thereby include the VDAC as a possible candidate for executing the pathogenic effects in PD brain.

## 4. Amyotrophic Lateral Sclerosis and VDACs

The progressive adult-onset neurodegenerative disease amyotrophic lateral sclerosis (ALS; also called Lou Gehrig’s disease) is based on the loss of upper and lower motor neurons in the spinal cord and brain [259]. Typically, the onset occurs at the age of 50 to 60 years, characterised by progressive paralysis, and finally these patients will die within 2–5 years upon onset by respiratory failure [260]. Curative treatments are not available for ALS so far [261]. While 90% of ALS cases are sporadic (sALS) without any genetic background, the remaining 10% of cases show an autosomal dominant trait (familial ALS, fALS). In more than three decades of research, over 40 different ALS-associated genes have been identified, among them for example copper–zinc superoxide dismutase (SOD1), transactive response DNA-binding protein 43 (TDP-43), and chromosome 9 open reading frame 72 (C9orf72) [262]. ALS shows several cellular deficits such as oxidative stress, mitochondrial dysfunction, glutamate excitotoxicity, axonal transport dysregulation, endosomal and vesicular transport impairment, aberrant RNA metabolism, and impaired protein homeostasis [263]. Mutations in the gene coding for the cytoplasmic SOD1 are associated with 20% of the fALS [264]. Disturbances in the redox properties of SOD1 yield atypical structural changes of the SOD1 protein, resulting in a gain of toxic properties, which mediates cell death of motor neurons in both sALS and fALS [265]. As shown in mouse motor-neuron-like NSC-34 cells, mutated SOD1 misfolds and accumulates in the cytosol, while there is a correlation between the mutation severity and the degree of protein misfolding. In addition, the mutation severity of SOD1 correlates with its property to associate with mitochondria [266]. This association of misfolded SOD1 with the OMM results in mitochondrial dysfunction and further on in cellular toxicity via two different mechanisms: First, misfolded SOD1 binds to mt-VDAC-1 at the OMM and changes its channel conductance for adenine nucleotides, disturbing the cellular energy supply [267]. Second, the import of proteins into mitochondria is suppressed by misfolded SOD1, yielding a changed protein composition [268]. The mutants of human SOD1 G93A and SOD1 H46R interact with the cytoplasmic part of mt-VDAC-1. This interaction was observed with reconstituted purified components and on isolated mitochondria derived from the spinal cord of an ALS rat model expressing mutant human SOD1. Spinal cord mitochondria from these ALS rats show reduced ADP passage through the OMM. As shown by a reconstitution experiment using a lipid bilayer, the channel conductance was inhibited by direct binding of mutant SOD1 to VDAC-1. Using a peptide array, the residues 28–61 of SOD1 were identified for the interaction with VDAC-1 [269]. Furthermore, mice expressing SOD1 G37R showed reduced VDAC-1 activity and an accelerating onset of fatal paralysis, yielding a reduced life span [267]. Moreover, there is an interaction between mutant SOD1 G93A and Bcl-2, altering the interaction between Bcl-2 and VDAC-1 and resulting in a reduced OMM permeability. The complex formation between SOD1 G93A and Bcl-2 can be inhibited by small SOD1-like peptides, preventing mitochondrial hyperpolarisation and mediating protection from cell death as demonstrated in NSC-34 cells [270]. A further study using the SOD1-G93A mouse model revealed that the interaction of mutant SOD1 with Bcl-2 induces a conformational change of Bcl-2, uncovering its toxic Bcl-2 homology 3 (BH3) domain and thereby converting it into a toxic protein [271]. The interaction of SOD G93A and SOD1 G85R with VDAC-1 could be narrowed down to the N-terminus of VDAC-1. Interestingly, wild-type SOD1 does not interact with VDAC-1. Expression of SOD1 G93A or SOD1 G37R, respectively, in NSC-34 cells and expression of SOD1 G93A in mouse embryonic stem cell-derived motor neurons resulted in cell death, which could be reduced upon the application of small cell-penetrating peptides, mimicking the VDAC-1 N-terminus. These VDAC-1 N-terminal-derived peptides interact with mutant SOD1, preventing the interaction with mt-VDAC-1 and thus protecting from mitochondrial dysfunction [272].

While under physiological condition HKI binds to mt-VDAC-1 and protects from apoptosis [99,100]; mutant SOD1 G93A competes with HKI for the same binding site on mt-VDAC-1 and consequently this SOD1-VDAC-1 association mediates mitochondrial dysfunction. Interestingly, application of peptides derived from the N-terminus of HKI recovered the cell viability and resulted in an increased mt-VDAC-1 expression and reduced mutant SOD1 accumulation at the mitochondria in NSC-34 cells expressing SOD1 G93A [273,274]. A proteomic approach was applied on NSC-34 cells, investigating how the proteome changes upon the expression of SOD1 G93A. The results showed changes in several mitochondrial proteins, among them a reduced expression of mt-VDAC-1 and mt-VDAC-2. In addition, changes of the post-translational modifications of mt-VDAC-2 were found, suggesting its involvement in regulation of apoptosis and mutant SOD1-mediated neurotoxicity [275]. A further study revealed changes in the post-translational modifications of VDAC-1 derived from NSC-34 cells expressing SOD1 G93A, showing that VDAC-1 has selective deamidations of asparagine and glutamine as well as over-oxidation of methionine and cysteines. These altered post-translational modifications may result in important structural changes of mt-VDAC-1 influencing the energetic metabolism of motor neurons in ALS [276].

Recently, in NSC-34 cells expressing human SOD1 G93A, changes of the post-translational modifications of VDAC-3 were found such as succination events, deamidation, and over-oxidation. In more detail, VDAC-3 with deamidation of asparagine 215 embedded in artificial membranes showed altered single-channel behaviour. This might affect the protective property of VDAC-3 against ROS, which is essential in the context of ALS [277]. An ALS mouse model expressing SOD1 G93A was treated with the VDAC inhibitor olesoxime, resulting in delayed muscle denervation and delayed death of motor neurons [278]. The administration of olesoxime was tested in a clinical phase II–III trail in ALS patients but it failed to show beneficial effects [279]. As recently demonstrated, mitochondria isolated from spinal cord of mutant SOD1 G93A rats and mice, respectively, showed oligomerisation of VDAC-1. The small molecule VBIT-12 is an inhibitor for VDAC-1 oligomerisation and thereby prevents apoptosis, like VBIT-4 as mentioned above (see 2). Administration of VBIT-12 in mutant SOD1 G93A mice improved muscle endurance, although the survival was not extended [269].

Interestingly, spinal motor neurons derived from the SOD1 G93A mouse model showed an increased level of VDAC-1 before the onset of symptoms [269]. An upregulated VDAC-1 expression due to an adeno-associated virus (AAV) 2/5 injected into the spinal cord of pre-symptomatic neonatal pups of SOD1 G93A mouse restored the mitochondrial respiratory profile. This can be explained by enhanced activity of key regulators of mitochondrial maintenance and function such as sirtuins (Sirt), the respiratory chain complex I and the receptor subunit of the TOM complex, Tom20 [280]. Recently, blood samples from ALS patients were used for analysing transcriptomic data with the help of an ARACNe-AP (Algorithm for the Reconstruction of Accurate Cellular Networks—Adaptive Partitioning) algorithm, revealing four subnetworks of hub genes including one subnetwork, with VDAC-3 being one hub gene. Each hub gene showed a connection to p53-mediated pathways, which might be linked to ALS neuroinflammation [281].

Another protein involved in ALS is the heterogeneous nuclear ribonucleoprotein TDP-43. A mouse model of mutant TDP-43 and induced pluripotent stem cells (iPSCs)-derived motor neurons from ALS patients showed that TDP-43 induced the release of mtDNA into the cytosol through the PTP. This could be prevented by VBIT-4, the VDAC-1 oligomerisation inhibitor [282], because mtDNA can be released through a channel of oligomeric VDAC-1 into the cytosol [83]. Furthermore, knockout of VDAC-1 in mouse embryonic fibroblasts (MEFs) overexpressing TDP-43 inhibited the expression of the innate immune-related factors Ifnb1 (Interferon Beta 1) and TNF [282]. A direct interaction between TDP-43 and VDAC-1 was shown by a proteomic screen investigating mitochondrial-interacting proteins of TDP-43 in a mouse model of motor neuron disease [283]. These studies suggest, regarding ALS pathogenesis, that TDP-43 may mislocalise into the mitochondria and there interact with VDAC-1, and thus enables the release of mtDNA into the cytosol.

As mentioned above (see 3), there is a protein complex built of VDAC-1 and GRP75 and further proteins for the crosstalk between ER and mitochondria at the MAM [258]. Recently, mechanisms of early ER stress were investigated using iPSCs-derived motor neurons from C9orf72-ALS/FTD patients who had a monogenic form of ALS due to a hexanucleotide repeat expansion in their C9orf72 gene, along with symptoms of frontotemporal dementia (FTD). These patients’ derived motor neurons showed elevated levels of GRP75 and VDAC-1, respectively, along with increased IP3R-VDAC-1 interaction [284]. In addition, studies of the spinal cords of C9-500 mice showed an increase in the IP3R-VDAC-1 interaction at post-natal day 125 (P125) and a decrease at P240 correlating to the GRP75 expression level. Furthermore, following AAV-forced expression of GRP75 in C9-500 mice protected from ER stress, mitochondrial function was normalised and the IP3R-VDAC-1 interaction increased [284]. In conclusion, neurons in C9orf72-ALS/FTD are vulnerable to ER–mitochondrial dysfunction and the critical endogenous GRP75 protein shows neuroprotective properties influencing the IP3R-VDAC-1 interaction.

Taken together, all three isoforms of VDAC are involved in ALS, although most studies focus on VDAC-1. ALS-related proteins such as mutated SOD1 and TDP-43 directly interact with mt-VDAC-1, while there are no studies showing any functional connection of pl-VDAC-1 in the pathogenesis of ALS. The interaction of mutated SOD1 with mt-VDAC-1 changes the channel conductance, resulting in disturbed cellular energy. Although the application of small peptide, inhibitors such as VBIT-4, VBIT-12, or olesoxime mediated beneficial and promising effects in the ALS models, unfortunately no drug has arisen for a treatment of ALS. As more than 40 genes are associated with ALS; maybe further ALS-relevant, VDAC-interacting proteins might be discovered in future.

## 5. Huntington’s Disease and VDACs

The fatal human neurodegenerative disorder Huntington’s disease (HD) is caused by a CAG repeat expansion in exon 1 of the huntingtin gene (HTT). HD is inherited in an autosomal dominant manner. Several CAG repeats are translated into N-terminal polyglutamine residues, resulting in a mutant HTT protein forming aggregates in different cells including neurons [285]. Aggregates of mutant HTT protein induce atrophy in subthalamic nuclei and in the basal ganglia, aberrations in the subcortical white matter, and finally result in the development of neurologic symptoms [286]. These symptoms comprise involuntary movements, such as dystonia, chorea, bruxism, bradykinesia, and rigidity as well as psychiatric symptoms along with cognitive decline resulting in dementia [287]. Different stages of disease are characterised by several complex cascades involving impaired proteostasis, excitotoxicity, oxidative stress, mitochondrial dysfunction, transcriptional dysregulation, and neuroinflammation [288]. So far, there is no therapy available to treat HD. Worldwide, 2.7 per 100,000 persons are affected by HD, while Europe shows a higher rate with 10 per 100,000 individuals [285].

The effect of olesoxime was investigated in the BACHD rat model of HD, in which full-length HTT along with 97 CAA/CAG repeats are overexpressed. The administration of olesoxime reduced cleavage of mutant HTT, accumulation of mutant HTT fragments, and calpain activation and resulted in improved mitochondrial function. Besides these neuropathological improvements, olesoxime treatment resulted in behavioural improvements as well. In respect to VDAC, olesoxime treated BACHD rats showed increased expression of mt-VDAC-1 and mt-VDAC-2 in the cortex and striatum, which was not observed in BACHD rats without olesoxime treatment [289]. In a further study, the HD mouse model R6/2 which expresses mutant HTT with 120 to 128 glutamines was investigated to assess mitochondrial function and the rate of oxygen consumption in vivo. The striatum showed a reduced oxygen consumption because of fewer mitochondria while the cortical oxygen consumption was lowered by disturbances of energetic pathways. Several mitochondrial proteins were compared in their expression level with wild-type mice and thereby a lower mt-VDAC-1 expression was found in the striatum, while there was no change in the cortex [290]. In line with this, mouse striatum-derived STHdh cells expressing mutant HTT with 111 glutamine residues, either homozygous or heterozygous, were investigated regarding their mitochondrial morphology, the mitochondrial disulfide relay system, and their function in HD. Besides several other findings, these cells showed a reduced VDAC expression, while this reduction was stronger in cells homozygously expressing mutant HTT than in heterozygously expressing cells [291]. Furthermore, the R6/2 mouse model was analysed by a proteomic approach, showing that six proteins were oxidised over the course of disease, among them VDAC-1 [292]. In order to study the role of the VDAC in the pathogenesis of HD, the VDAC was extracted from PC12 model cells expressing HTT or mutant HTT with 74 repeats of glutamine respectively. The VDAC was reconstituted into artificial membranes for further analysis. Although the ratio between the three VDAC isoforms were not changed, only VDAC-1 showed a changed open state conductance and a changed voltage-dependence upon the expression of mutant HTT but not of wild-type HTT expression [293]. A recent study showed that extracellular vesicles derived from HD cells contained mtDNA and several mitochondrial proteins, among them VDAC-1. These HD cells were neurons with a striatal phenotype obtained by reprogramming of neuronal stem cells derived from iPSCs, which were derived from reprogrammed human fibroblasts of individuals considered as HD carriers. Stress induces mitochondria to generate mitochondrial-derived vesicles, which fuse with the endolysosomal system, forming multivesicular bodies that are released as extracellular vesicles from the cells [294]. Recently, rats were treated with the HD model substance 3-nitropropionic acid (3-NP), resulting in impaired mitochondrial respiration and mitochondrial dynamics as well as in induced ER stress. Proteins such as VDAC-1 and Grp75, which are involved in the mitochondria–ER communication (see 3), showed elevated expression levels. Application of bezafibrate inhibited these 3-NP-mediated changes, including a normalised expression level of VDAC-1 and Grp75 [295]. In a recent study, rats were treated with 3-NP and then tested how the antioxidant flavonoid morin hydrate (MH) may counteract the 3-NP-mediated HD phenotype. MH showed neuroprotective properties because motor dysfunction, degeneration of striatal neuros, ER stress, apoptosis, and mitophagy were reduced. Thereby, MH mediated inactivation of VDAC-1 by its phosphorylation [296].

Taken together, data from different cellular and animal models show an involvement of VDAC-1 and VDAC-2 in HD. Substances such as olesoxime, bezafibrate, and MH mediated beneficial effects towards HD in these model systems and thereby involved the VDAC directly or indirectly. However, there are currently no data available about the expression level of the VDAC in HD patients. Furthermore, no functional relationship between the plasmalemmal localised VDAC and HD has been demonstrated so far.

## 6. Further Neurodegenerative Conditions and VDACs

The neurodegenerative lysosomal storage disease neuronal ceroid lipofuscinosis (NCL)—also known as Batten disease—comprises a group of 13 subtypes of NCL. Distinct genes are associated with each NCL subtype, having mutations coding for lysosomal enzymes, transmembrane proteins, or secretory proteins. This autosomal-recessive disorder manifests in infants by symptoms such as vision impairment, epilepsy, motor and cognitive dysfunctions, and premature death. Although, there are different treatment approaches such as pharmacological therapy, enzyme replacement therapy, stem cell therapy, and gene therapy, all approaches have shown limited efficacy so far [297]. In one study, two different genetic mouse models of NCL were investigated, showing a progressive breakdown of synapses and axons in the cortex and thalamus of these mice. The expression levels for a group of proteins involved in axonal and synaptic vulnerability were quantified, demonstrating individual expression profiles. Interestingly, the two proteins’ pituitary tumour-transforming gene 1 (PTTG1) product and VDAC-1 showed significant changes in their expression level in the thalamus at the time points of pre- or early onset of symptoms in both mouse lines. Therefore, VDAC-1 was suggested to be used as an early biomarker for NCL [298].

Wernicke’s encephalopathy (WE) is a neurological disorder caused by thiamine (vitamin B1) deficiency, characterised by eye movement abnormalities, acute confusion, and ataxia [299]. WE can develop into a chronic syndrome called Wernicke–Korsakoff syndrome (WKS) [300]. One study showed the investigation of a WKS rat model regarding thiamine deficiency on spatial learning and differential protein expression. The expression level of seven thalamic proteins was significantly changed, among them the VDAC. The VDAC expression was decreased when the rats showed worse performance in the behavioural test, called the Morris Water Maze. While the VDAC expression was decreased on the protein level, the mRNA level was unchanged, suggesting that post-transcriptional processes may result in decreased expression [301].

Recently, old male mice showed reduced length and area of their hippocampal mitochondria, increased neurodegeneration, a decline in recognition memory, and changed protein expression, including a decline in VDAC-1 in the hippocampus [302].

## 7. Conclusions

**Figure 5:**
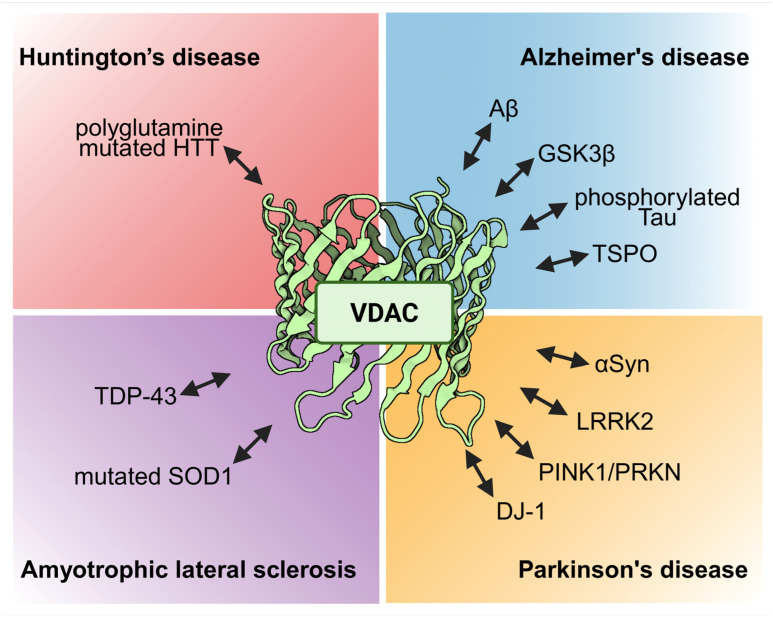
Overview of disease-specific proteins interacting with the VDAC of the discussed neurodegenerative diseases. The VDAC is symbolised by the three-dimensional structure of human VDAC-1 (Protein Data Bank entry: 2JK4; [20]). This figure was created in https://BioRender.com/txidazd (accessed on 19 May 2025).

In summary, the VDAC is involved in neurodegenerative diseases such as AD, PD, ALS, and HD as well as additional neurodegenerative conditions. Typically, VDAC physically interacts with disease-specific proteins such as Aβ, GSK3β, phosphorylated tau, TSPO, αSyn, LRRK2, PINK1/PRKN, DJ-1, mutated SOD-1, TDP-43, and polyglutamine-mutated HTT, resulting in modulation of mitochondrial permeability and participating in apoptosis (Figure 5). Furthermore, the expression level of the VDAC might also be regulated by miRs like miR-29a and miR-7 or changed upon disease conditions. In line with the abundance of the different VDAC isoforms, most studies deal with VDAC-1, while investigations on VDAC-2 or VDAC-3 are less numerous. Although the VDAC is a prominent protein of the OMM, it is found in other cellular locations, e.g., in the plasma membrane. Only AD showed an involvement of pl-VDAC-1, while for all other neurodegenerative diseases, reports are not available so far. Furthermore, there are no reports up to now that any mutation of VDAC itself would lead to any neurodegenerative diseases. The VDAC might be an interesting target for developing treatment options, as evidenced by several inhibiting interventions using small molecules such as VBIT-4 and VBIT-12, olesoxime, resveratrol, idebenone, bezafibrate, morin hydrate, application of interfering peptides, antibodies, or metformin, the latter being an established drug for the treatment of T2D. While still in early stages of research, T-cell therapy is being explored as a potential therapeutic approach for neurodegenerative conditions like AD and PD [303]. This may include targeting novel specific proteins such as the VDAC involved in neuronal damage. Taken together, the VDAC may turn out to be a major player in regulating neuronal survival in neurodegenerative diseases and, therefore, may provide an emerging platform for so far unrecognised therapeutical treatment options.

## Figures and Tables

**Table 1:** Comparison of some selected features among the three VDAC isoforms.

Feature	VDAC-1	VDAC-2	VDAC-3
Exons in mammals	9	10	9
Gene encoded in nucleus	yes	yes	yes
Splice variants	2	1	1
cDNA homology to VDAC-1	100%	90%	68%
Expression level	Highest expression level	Lower expression	Lowest expression
Tissue distribution	Brain Heart Liver Skeletal muscles	Brain Heart Liver Skeletal muscles	Liver Lung Spleen Ovary adrenal gland Testis
Three-dimensional structure solved	For human and mice	For zebrafish	No structure so far
Conductance in artificial membranes in vitro	High	High	Low
Extramitochondrial locations	Sarcoplasmic reticulum Endoplasmic reticulum Plasma membrane	Plasma membrane acrosomal membrane of spermatozoa Outer dense fibres of sperm flagellum	Outer dense fibres of sperm flagellum
